# Validation of Progression-Free Survival Rate at 6 Months and Objective Response for Estimating Overall Survival in Immune Checkpoint Inhibitor Trials: A Systematic Review and Meta-analysis

**DOI:** 10.1001/jamanetworkopen.2020.11809

**Published:** 2020-09-08

**Authors:** Peey-Sei Kok, Doah Cho, Won-Hee Yoon, Georgia Ritchie, Ian Marschner, Sally Lord, Michael Friedlander, John Simes, Chee Khoon Lee

**Affiliations:** 1 National Health and Medical Research Council Clinical Trials Centre, University of Sydney, Sydney, Australia; 2 Macarthur Cancer Therapy Centre, Campbelltown Hospital, Sydney, Australia; 3 Cancer Care Centre, St George Hospital, Sydney, Australia; 4 Mid North Coast Cancer Institute, Port Macquarie Base Hospital, Port Macquarie, Australia; 5 School of Medicine, University of Notre Dame, Sydney, Australia; 6 Nelune Cancer Centre, Prince of Wales Hospital, Sydney, Australia

## Abstract

**Importance:**

Progression-free survival (PFS) rate at 6 months has been proposed as a potential surrogate for overall survival (OS) rate at 12 months for immune checkpoint inhibitor (ICI) trials but requires further assessment for validation.

**Objective:**

To validate 6-month PFS and objective response rate (ORR) as estimators of 12-month OS in the ICI arms of randomized clinical trials (RCTs).

**Data Sources:**

Electronic databases (Medline, EMBASE, and the Cochrane Central Register of Controlled Trials) were searched for ICI RCTs published between January 2000 and June 2019.

**Study Selection:**

Eligible studies were phase 2 and phase 3 ICI RCTs in advanced solid cancers that reported ORR, PFS, and OS. A total of 99 articles (from 60 studies) of 2502 articles were selected by consensus.

**Data Extraction and Synthesis:**

Data were screened and extracted independently. Estimation models for 12-month OS and to assess correlation coefficient between end points were developed using linear regression. Data were extracted in July 2019, and analyses were conducted in September 2019. This study is reported following the Preferred Reporting Items for Systematic Reviews and Meta-analyses (PRISMA) reporting guideline.

**Main Outcomes and Measures:**

Validation of previously reported 6-month PFS and ORR estimation models for 12-month OS using contemporary RCTs. Calibration of 6-month PFS and ORR model-estimated vs observed 12-month OS in ICI arms were assessed by correlation coefficient (*r*) and weighted Brier scores. Secondary analyses were performed for subgroups (ie, ICI-only, ICI-combination, line of therapy, programmed cell death 1 ligand 1 selected, and unselected).

**Results:**

Data from 60 RCTs with 74 experimental ICI arms were used. The development data set included 25 arms from studies published January 2000 to January 2017. The estimation model for 12-month OS using 6-month PFS was: (1.06 × PFS6) + 0.16 + (0.04 × melanoma) − (0.03 × NSCLC) + (0 × other tumors), in which *PFS6* indicates 6-month PFS and *NSCLC* indicates non–small cell lung cancer. The estimation model for 12-month OS using ORR was (0.15 × ORR) + 0.52 + (0 × melanoma) − (0.02 × NSCLC) − (0.01 × other tumors). A total of 49 arms from studies published after January 2017 to June 2019 formed the validation data set. When the models were applied on the validation data set, calibration between the 6-month PFS model estimated vs observed 12-month OS was good (*r* = 0.89; Brier score, 0.008), but poor for the ORR model (*r* = 0.47; Brier score, 0.03). Findings were similar across all subgroups.

**Conclusions and Relevance:**

The findings of this study suggest that the estimation model using 6-month PFS could reliably estimate 12-month OS in ICI trials. This study could assist in better selection and prioritization of ICI agents for testing in RCTs based on phase 2 single-arm RCT results.

## Key Points

**Question:**

Can surrogate end points, such as progression-free survival, be used to estimate overall survival in immunotherapy trials?

**Findings:**

This systematic review and meta-analysis using data from 60 published immunotherapy randomized clinical trials in advanced solid cancers found that 6-month progression-free survival in the immunotherapy arm estimated 12-month overall survival well using a statistical model that accounts for tumor type.

**Meaning:**

These findings suggest that this model could assist with selecting immunotherapy agents for further testing in large randomized clinical trials and with regulatory approvals in rare cancers for which randomized trials are not feasible.

## Teaser

This systematic review and meta-analysis examines models using 6-month progression-free survival or objective response rate to estimate overall survival at 12 months in immune checkpoint inhibitor (ICI) randomized clinical trials.

## Introduction

The number of clinical trials testing the efficacy of immune checkpoint inhibitors (ICIs) targeting programmed cell death 1 (PD-1), programmed cell death 1 ligand 1 (PD-L1) and cytotoxic T-lymphocyte–associated antigen 4 (CTLA-4) has increased exponentially. Since the first ICI study was initiated in 2006 to evaluate the PD-1 monoclonal antibody nivolumab, more than 2000 ICI trials actively recruited more than 380 000 volunteers between 2006 and 2018.^1^ While early trials commonly tested an anti–PD-1 or anti–PD-L1 agent as monotherapy or doublet therapies, there is an increasing number of trials investigating ICI in combination with chemotherapy or molecularly targeted therapies, such as tyrosine kinase inhibitors, PARP inhibitors, or angiogenesis inhibitors. There is optimism that ICIs will improve overall survival (OS) in a wide range of cancers; hence, there is a high level of interest in ICI trials.

Regulatory drug approval is based on substantial evidence of safety and efficacy measured using clinically important end points, such as progression-free survival (PFS) and OS, and supported by quality of life analyses in phase 3 randomized clinical trials (RCTs).^2,3^ However, such studies usually require large sample sizes and can take years to report mature OS results. There is a need for intermediate surrogate end points that estimate treatment benefit and could be used in submissions for accelerated regulatory approval. To date, the most widely used surrogate end points have been objective response rate (ORR) and PFS, and they are commonly measured in most clinical trials. Specifically for ICIs, many have already been granted accelerated approval, with some based on the results from nonrandomized single-arm or basket trials with ORR or PFS as primary end points.^4,5,6,7^

There is ongoing concern about the validity of using improvements in ORR and PFS to infer impact of treatment on OS and adoption of costly new therapeutics into clinical practice based on surrogate end points alone.^8,9^ For estimation of relative treatment effectiveness, ORR odds ratios and PFS hazard ratios (HRs), used widely as primary trial end points in cancer trials, have been shown to have only moderate to poor correlation with OS HRs.^8^ In addition, treatment effect sizes from phase 2 RCTs often overestimate effect sizes when compared with phase 3 RCTs. Liang et al^10^ reported that relative treatment effects on PFS were 26% larger in phase 2 RCTs than the matched phase 3 RCTs with the same experimental treatment.^11^ Overestimations in the magnitude of treatment effects on PFS are also observed in ICI RCTs.^12,13^

While the focus of research on surrogate end points for trials has largely been on the validity of these end points to estimate relative treatment effects on OS in phase 3 RCTs, there is a more fundamental need to identify valid surrogate end points to select phase 2 RCTs, to be investigated further in phase 3 RCTs. We have previously proposed 6-month PFS as a practical surrogate for 12-month OS in ICI trials of advanced solid organ cancers using a model that adjusted for tumor type.^14^ We have previously shown that ORR correlated poorly with 12-month OS. Limited validation of this approach was performed using small single-arm studies.^14^

The aim of this systematic review and meta-analysis is to validate 6-month PFS and ORR as estimators of OS in a much larger data set of more contemporaneous RCTs. We also examined their validity based on subgroups of newer ICI combination strategies, line of treatment, and PD-L1 enrichment.

## Methods

All study data are publicly available from the published trials. Additional ethics approval was not required for this analysis. We followed the Preferred Reporting Items for Systematic Reviews and Meta-analyses (PRISMA) reporting guidelines for systematic reviews and meta-analyses. We performed a systematic search for eligible RCTs published between January 2000 and June 2019 using Medline, EMBASE, and the Cochrane Central Register of Controlled Trials. We also manually searched conference abstracts, posters, and presentations from websites of the American Society of Clinical Oncology and European Society of Medical Oncology. Search terms included *atezolizumab*, *avelumab*, *nivolumab*, *pembrolizumab*, *ipilimumab*, *tremelimumab*, *CTLA-4*, *PD-1*, *PD-L1*, *checkpoint inhibitor*, and *randomized trial*.

RCTs were eligible if they assessed anti–PD-1, anti–PD-L1, or anti–CTLA-4 ICIs in unresectable locally advanced or metastatic solid tumors and reported ORR, PFS, and OS. We excluded RCTs of ICIs in neoadjuvant, adjuvant, or maintenance settings and in stage III or resected stage IV cancers and RCTs assessing ICI in combination with radiotherapy or local injection therapies, different doses of the same ICI, local injection therapies or other novel ICIs not targeting anti–PD-1, anti–PD-L1, or anti–CTLA-4. We excluded small RCTs, defined as a sample size of 80 patients or fewer.

### Data Extraction

Two of us (P.-S.K and W.-H.Y.) screened the RCTs independently and extracted the following data: trial phase, sample size, treatment arms, key baseline characteristics, tumor type, line of therapy, PD-L1 status, and outcomes (ie, ORR, PFS, and OS). Landmark 6-month PFS was defined as proportion of patients who remained alive and progression-free at 6 months, whereas landmark 12-month OS was defined as proportion of patients who remained alive at 12 months. We extracted 6-month PFS and 12-month OS for both ICI (experimental) and control arms from the Kaplan-Meier curves using Digitizelt software version 2.0 if they were not reported in the study. Any discrepancies were resolved by consensus.

### Statistical Analysis

#### Twelve-Month OS Estimation

The primary objective of the study is to validate 6-month PFS and ORR as estimators of 12-month OS. We used linear regression to develop 6-month PFS and ORR models to estimate 12-month OS adjusting for tumor type (ie, melanoma, non–small cell lung cancer [NSCLC], or other). The model development data set consisted of the ICI containing experimental treatment arms from RCTs identified from our previous literature search from January 2000 to January 2017.^14^ The model validation data set consisted of RCTs published from January 2017 to June 2019. eTable 1 in the Supplement outlines the models using ORR and 6-month PFS to estimate 12-month OS developed from the training data set.^14^

We validated the performance of the 6-month PFS and ORR models to estimate 12-month OS in the validation data set by visually assessing the observed vs model-estimated 12-month OS on a calibration plot, calculating the correlation coefficient (*r*) and the Brier score (the mean squared difference between the observed and estimated values weighted by sample size of each ICI arm).^15^ The lower the Brier score, the more accurate the model estimations. A well-performing estimation model is defined by strong correlation between estimated and observed 12-month OS (ie, *r* close to 1) and a low Brier score (ie, close to 0).

#### Secondary Objectives

##### Subgroup Analysis

We further assessed the performance of each model in ICI treatment and trial population subgroups in the validation data set using the same approach. The subgroups included ICI-only, combination of ICI and chemotherapy or tyrosine kinase inhibitors, first line, second or subsequent line, PD-L1 enriched, and PD-L1 unselected trial samples.

##### Refining the ORR Model

Our prior study found poor correlation between ORR and 12-month OS, and the ORR model poorly estimated 12-month OS in single-arm studies.^14^ In this study, we assessed the correlation among ORR, 6-month PFS, and 12-month OS in the entire (ie, development and validation) RCT data set. We attempted to improve the ORR model performance by assessing the correlation between ORR and 12-month OS in our 6 subgroups and refitting the model in the subgroup with the strongest correlation. We used the ordinary least-squares regression approach to re-estimate β coefficients of the covariates and weighted the model according to sample size of the ICI arms.

##### Correlation With Relative Treatment Effects

We also plotted the correlation between PFS HRs and ORR risk ratios vs OS HRs using data from both treatment and control arms. The strength of correlations was again expressed as the correlation coefficient, *r*.

We used STATA statistical software version 15 (StataCorp) for all linear regression analyses weighted by sample size, which were used to develop the models.^14^ The same linear regression was applied in separate groups (subgroup analyses) to evaluate the estimation of 12-month OS. Data were extracted in July 2019, and analyses were conducted in September 2019.

## Results

### Search Results

**Figure 1.**
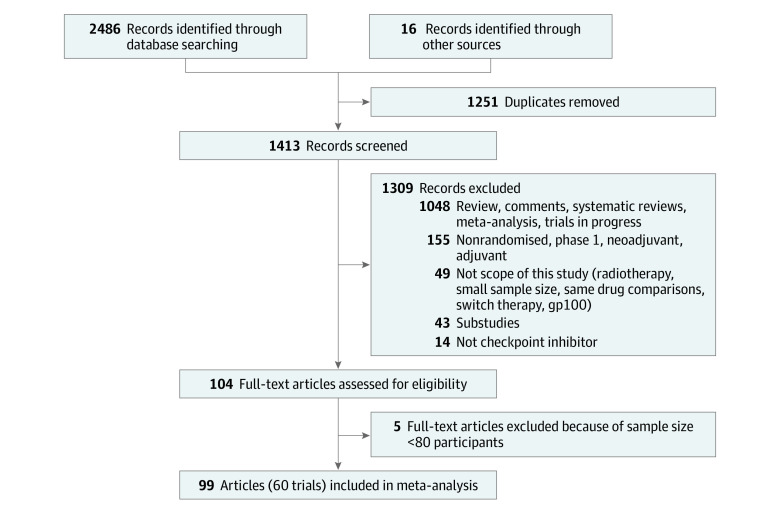
Study Selection Flowchart

The search strategy (Figure 1) identified 60 eligible RCTs, including 14 multi-arm studies (eTable 2 in the Supplement), including 74 ICI experimental arms with 17 891 patients. The ICIs studied included 21 arms examining pembrolizumab, 17 arms examining nivolumab, 12 arms examining ipilimumab, 7 arms examining durvalumab, 6 arms examining tremelimumab, 15 RCTs examining atezolizumab, and 3 arms examining avelumab (eTable 2 in the Supplement). Most ICI arms were extracted from phase 3 RCTs (56 arms [76%]), ICI-only (51 arms [69%]), first-line settings (40 arms [54%]) conducted in PD-L1 unselected populations (52 arms [70%]). The included studies were conducted in patients with NSCLC (27 arms [36%]), melanoma (12 arms [16%] and other tumor types (35 arms [47%]) (eTable 3 in the Supplement). Twenty-five treatment arms formed the model development data set for the 6-month PFS and ORR models, and 49 treatment arms formed the validation data set. Minimum follow-up reported in these studies ranged from 4 to 48 months, with a median of 13 months.

### Estimation of 12-month OS

**Figure 2.**
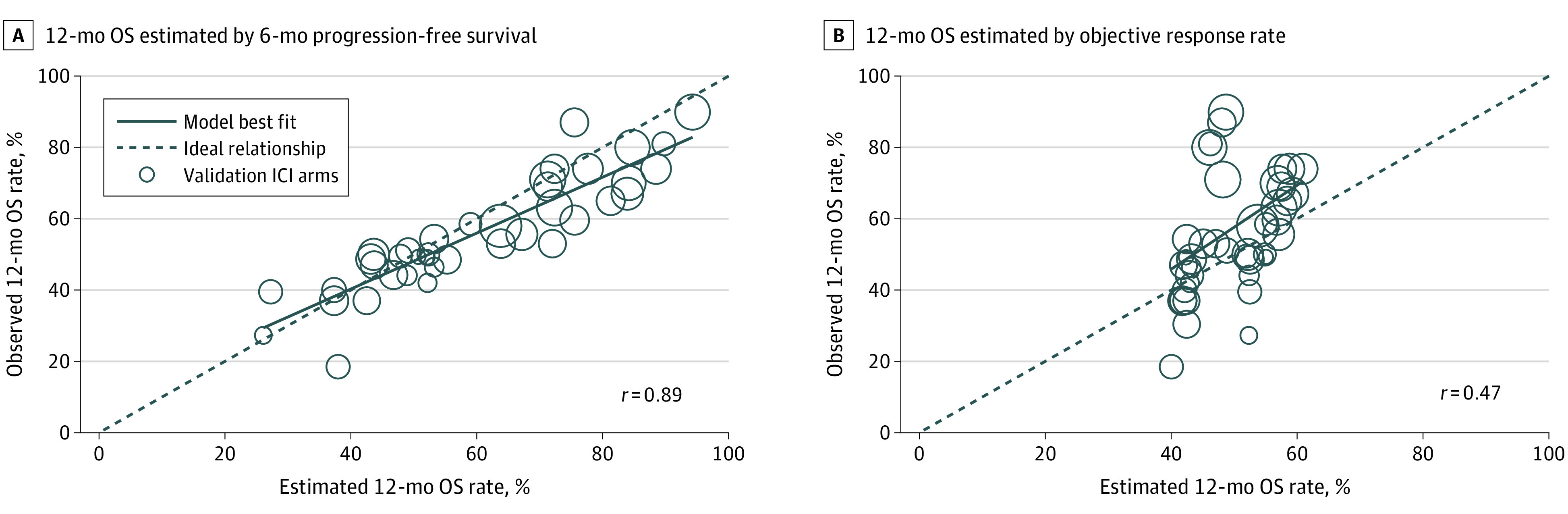
Estimated vs Observed 12-Month Overall Survival (OS) Rate Each circle indicates a study from the validation data set; circle sizes, sample size of immune checkpoint inhibitor (ICI) arm of the study.

The estimation model for 12-month OS using 6-month PFS was (1.06 × PFS6) + 0.16 + (0.04 × melanoma) − (0.03 × NSCLC) + (0 × other tumors), in which *PFS6* indicates 6-month PFS, and the 12-month OS model using ORR was (0.15 × ORR) + 0.52 + (0 × melanoma) − (0.02 × NSCLC) − (0.01 × other tumors) (eTable 1 in the Supplement). When applying the models to the ICI arms of the validation data set, the 6-month PFS model showed good calibration for estimation of 12-month OS (*r* = 0.89; Brier score 0.008) (Figure 2A). The 6-month PFS model also performed well in all ICI subgroups (eFigure 1 in the Supplement). The performance was particularly good for ICI-only arms (*r* = 0.94) (eFigure 1 in the Supplement).

The ORR model showed poor calibration for estimation of 12-month OS (*r* = 0.47; Brier score 0.03) (Figure 2B) in ICI arms that included both ICI combination and ICI-only studies in the validation data set. The ORR estimation model for 12-month OS also performed poorly in all subgroups (eFigure 2 in the Supplement).

### Correlations Among 6-month PFS, ORR, and 12-month OS in ICI Arms

**Figure 3.**
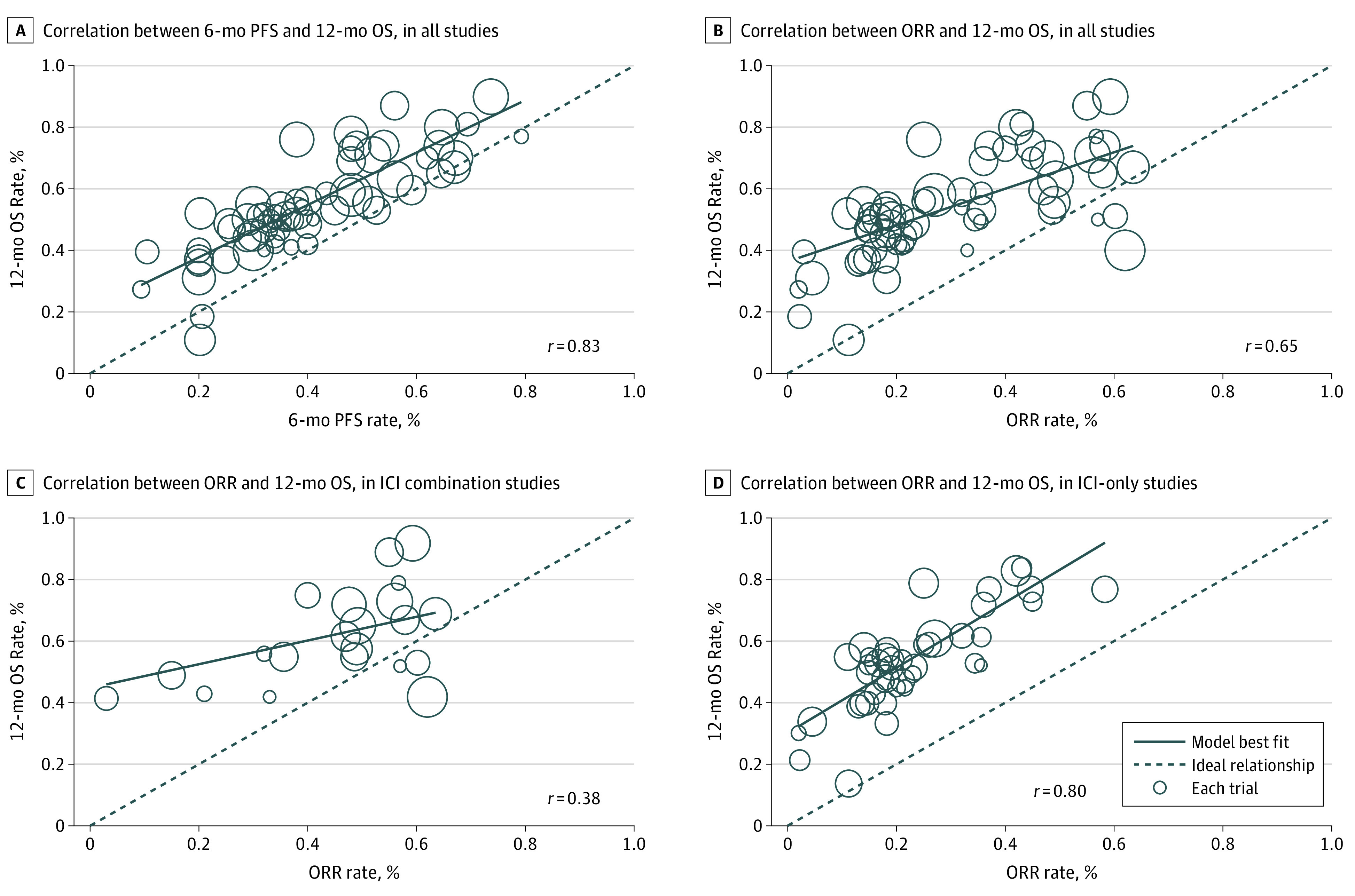
Correlation Between 6-Month Progression-Free Survival (PFS), Objective Response Rate (ORR), and 12-month Overall Survival (OS) in Immune Checkpoint Inhibitor (ICI) Arms Circle sizes indicate sample size of ICI arm of the study.

Other than estimation of 12-month OS, we also assessed the correlation coefficients among end points. Using data from 63 ICI arms, the correlation between 6-month PFS and 12-month OS was strong (*r* = 0.83) (Figure 3A). Using data from 64 ICI arms, the correlation was moderate between ORR and 12-month OS (*r* = 0.65) (Figure 3B).

### Correlations Among Relative Comparisons of Treatment Effects in All RCTs

**Figure 4.**
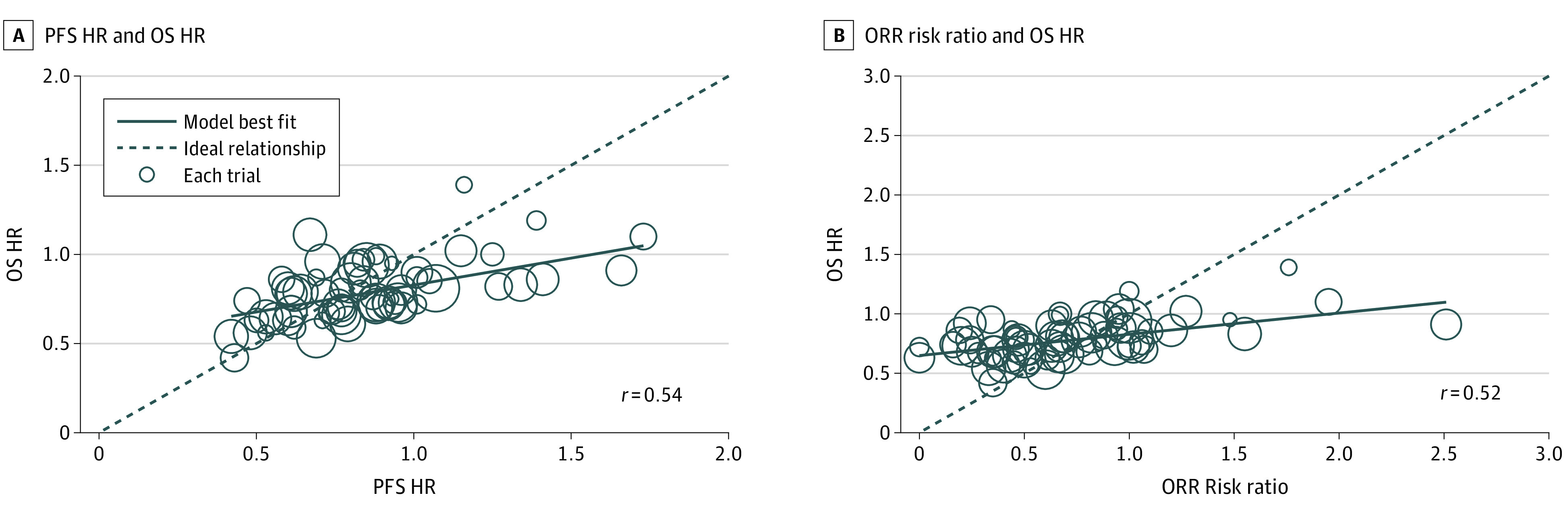
Correlation Among Treatment Effects in ICI RCTs HR indicates hazard ratio; circle sizes, total sample size of each randomized clinical trial; PFS, progression-free survival; ORR, objective response rate; and OS, overall survival.

Using data available from 65 RCTs (65 treatment comparisons of ICI vs control), the correlation between PFS HRs and OS HRs was moderate (*r* = 0.54) (Figure 4A). Using data from 66 RCTs, the correlation between ORR risk ratios and OS HRs was moderate (*r* = 0.52) (Figure 4B).

### Refining the ORR Model

In the subgroup analysis, the correlation between ORR and 12-month OS in the ICI-only arms was stronger (*r* = 0.80) than in the ICI combination (TKI or chemotherapy) arms (*r* = 0.38) (Figure 3C and D), and stronger in the second- or subsequent-line settings (*r* = 0.66) than first-line settings (*r* = 0.37) (eFigure 3 in the Supplement). Nearly all second- and subsequent-line studies (32 of 34 studies) were ICI-only trials (Table). In view of these findings, we refitted the ORR estimation model for 12-month OS using data from ICI-only treatment arms: (1.012324 × ORR) + 0.3006825 + (0 × melanoma) + (0.0021079 × NSCLC) – (0.038521 × other tumors).

**Table.** Frequency of Line of Treatment vs Type of ICI Studies

Treatment type	Treatment line, No (%)		Total, No.
	First	Second or subsequent	
ICI only	19 (37)	32 (63)	51
ICI combination	21 (91)	2 (9)	23
Total	40 (54)	34 (46)	74

Abbreviations ICI, immune checkpoint inhibitor.

Given our findings for strong correlation between 6-month PFS and 12-month OS in the entire data set, we did not attempt to refit the original 6-month PFS model (eTable 1 in the Supplement).

## Discussion

This systematic review and meta-analysis provides a 6-month PFS model that was used to estimate 12-month OS with good calibration in ICI arms of a larger validation data set and validates 6-month PFS as an estimator of 12-month OS. Consistent with our prior study,^14^ we confirm that ORR was a poor estimator of 12-month OS when ICI combination studies were included. In contrast, ORR estimates 12-month OS well within trials investigating ICIs alone. Thus, a new model to estimate 12-month OS based on ORR was generated for ICI-only studies.

Study data were derived from a comprehensive review of 74 ICI treatment arms from 60 contemporary ICI RCTs involving more than 17 000 patients, with ORR and PFS assessed by standardized criteria. The pooled data set consisted of a heterogeneous population testing 7 different ICI agents, from first- to fourth-line treatment, in PD-L1-enriched as well as unselected populations, and in 12 different tumor types. To avoid selection bias, we included both positive and negative trials. Our 6-month PFS for 12-month OS model was previously validated in 19 single-arm studies and now in 74 treatment arms from 60 RCTs conducted in diverse settings, widening its generalizability.

Current research examining validity of surrogate end points is predominately based on assessment of relative comparisons of treatment effects, such as HRs for PFS or OS, between randomized treatment arms.^16,17,18^ These studies have demonstrated poor correlations among PFS, ORR, and OS in chemotherapy and ICI trials.^16,17,18^ Using individual patient data submitted to the US Food and Drug administration for regulatory approval, a study by Mushti et al^16^ reported poor correlation among PFS HRs and ORR odds ratios with OS HRs in ICI trials, at both the trial level and patient level. Two other meta-analyses of ICI RCTs^17,18^ also reported that PFS HRs did not correlate with OS HRs. A 2019 study by Wang et al^18^ using a milestone restricted mean survival time ratio approach at different landmark times reported that the 6-month PFS ratios correlated poorly with OS HRs.

Our research differs from these studies, as we examined PFS as an estimator of OS within ICI arms. Our focus was not on relative comparisons between experimental and control arms. Our work used commonly reported surrogate end points, 6-month PFS and ORR, to develop statistical models to estimate 12-month OS adjusting for tumor type rather than using median PFS. We believe that our models are particularly applicable to single-arm phase 2 ICI RCTs, for which OS data may not be available, to estimate OS from observed 6-month PFS to facilitate decision-making to proceed with randomized phase 3 studies.

Assessment of relative comparisons of treatment effect on surrogate and OS outcomes is a challenge but remains an important area of research. Our study showed that 6-month PFS reliably estimated 12-month OS at treatment-arm level, but PFS HRs correlated only modestly with OS HRs. It is important to note that in relative comparison, the correlation between surrogate outcomes and OS relies not only on the performance of the experimental arm but also the control arm. The current approach may be problematic since the control arms are heterogeneous, with treatment types ranging from ICI, chemotherapy, targeted therapy, best supportive care, and even placebo. Interpreting results for the strength of surrogacy in relative treatment comparisons can be difficult, as performance of the control arms is expected to be different.

There are a number of reasons why our original ORR for 12-month OS model estimated 12-month OS poorly. This model was based on a small number of studies (ie, 25 ICI treatment arms) and, unlike the 6-month PFS for 12-month OS model, the correlation between ORR and 12-month OS in our previous study was poor (*r* = 0.08).^14^ Despite inclusion of an additional 49 treatment arms, correlation between ORR and 12-month OS was better but remained moderate (*r =* 0.63). This poor correlation is not specific to immunotherapy but has been consistently reported in chemotherapy trials of metastatic cancers.^8^ We demonstrated that the correlation between ORR and 12-month OS was stronger in ICI-only RCTs compared with the ICI combination RCTs. Almost all of these ICI-only RCTs were second- or subsequent-line studies; hence, fewer patients may have received effective subsequent treatment on disease progression. Across multiple tumors, chemotherapy after initial immunotherapy has been associated with improved and prolonged response than traditionally observed.^19,20^ In contrast, chemotherapy, when given before immunotherapy, may also upregulate PD-L1 and improve response to later-line ICIs.^21^ Thus, ICI used in combination with chemotherapy may alter the natural history of the disease compared with ICI-only treatment. We developed a new estimation model for 12-month OS based on reported ORR based on the ICI-only treatment arms and further research is ongoing in validating this new estimation model.

This study has several important implications. The ability to accurately estimate for OS from observed 6-month PFS using our model may allow smaller future ICI studies with shorter follow-up and earlier results saving resources. It may help better select and prioritize ICI agents, either as monotherapy or in combination with other treatments, for testing in phase 3 RCTs and reduce failure rates. Where phase 3 RCTs are not feasible owing to limitations, such as small sample size in rare cancers, 6-month PFS results that estimate for promising 12-month OS outcomes may assist regulators, policy makers, and funding bodies to better assess treatment efficacy even if evidence is limited to smaller, non-randomized trials. Importantly, our work may serve as a platform for future estimation models that incorporate multiple surrogate end points, including molecular surrogates and pharmacodynamic markers for a more sophisticated estimation of OS with ICI therapy.

### Limitations

There are some limitations to this study. We are unable to account for differences in follow-up time, which ranged from 4 to 48 months in our included studies. Although the landmark outcomes (ie, 6-month PFS and 12-month OS) were adjusted for censoring, we were not able to analyze the PFS and OS over the complete follow up. Thus, our conclusions are only relevant to the particular landmark times (ie, 6 months and 12 months). We were unable to assess the effect of subsequent therapies on OS, as these data were infrequently reported. Some studies reported primary end points based on both PD-L1–enriched and PD-L1–unselected populations. Our results included the intention-to-treat population whenever possible (mostly PD-L1 unselected); therefore, our findings may underestimate the outcome of PD-L1–selected studies. We included data from 17 RCTs reported only in the form of conference abstracts, posters, or oral presentations; we were unable to critically appraise the quality of these trials. Pseudoprogression, a unique phenomenon of ICIs, is thought to be underestimated by standard response evaluation criteria in solid tumors assessment and better captured by modified response evaluation criteria in solid tumors using immune-related response criteria^22^ or immunotherapy response evaluation criteria in solid tumors .^23^ Among the included RCTs in this study, only 1 RCT assessed outcome according to immune-related response criteria and none by immune response evaluation criteria in solid tumors. However, pseudoprogression is often a retrospective diagnosis and rare, estimated to occur in less than 10% of patients across a variety of tumor types,^24^ and will not likely impact on our results.

## Conclusions

The 6-month PFS for 12-month OS model developed in this systematic review and meta-analysis reliably estimated 12-month OS from 6-month PFS using a larger validation data set. A newly proposed ORR to 12-month OS model for ICI-alone studies has been developed, but validation of this model is still required.

## Notes

Supplement. **eTable 1.** Predictive Models Developed From Training Data Set to Estimate End Points **eTable 2.** Summary of Included Trials **eTable 3.** Summary of Immune Checkpoint Inhibitor Arms in Included Trials **eFigure 1.** Six-Month Progression Free Survival Model–Predicted vs Observed 12-Month Overall Survival in Immune Checkpoint Inhibitor Arms in the Subgroups and Validation Trial Data Set **eFigure 2.** Objective Response Rate Model–Predicted vs Observed 12-Month Overall Survival in Immune Checkpoint Inhibitor Arms in the Subgroups and Validation Trial Data Set **eFigure 3.** Correlations Between Objective Response Rate and 12-Month Overall Survival in Immune Checkpoint Inhibitor Arms in the Subgroups and Entire Trial Data Set **eReferences.**
