# Supplementary material for: Validation of Progression-Free Survival Rate at 6 Months and Objective Response for Estimating Overall Survival in Immune Checkpoint Inhibitor Trials: A Systematic Review and Meta-analysis
Source: JAMA Netw Open. 2020 Sep 8;3(9):e2011809. doi: 10.1001/jamanetworkopen.2020.11809 (PMC7489825; doi:10.1001/jamanetworkopen.2020.11809)
Supplement: Supplement. — eTable 1. Predictive Models Developed From Training Data Set to Estimate End Points eTable 2. Summary of Included Trials eTable 3. Summary of Immune Checkpoint Inhibitor Arms in Included Trials eFigure 1. Six-Month Progression Free Survival Model–Predicted vs Observed 12-Month Overall Survival in Immune Checkpoint Inhibitor Arms in the Subgroups and Validation Trial Data Set eFigure 2. Objective Response Rate Model–Predicted vs Observed 12-Month Overall Survival in Immune Checkpoint Inhibitor Arms in the Subgroups and Validation Trial Data Set eFigure 3. Correlations Between Objective Response Rate and 12-Month Overall Survival in Immune Checkpoint Inhibitor Arms in the Subgroups and Entire Trial Data Set eReferences. [file jamanetwopen-e2011809-s001.pdf]

Supplementary Online Content

Kok P-S, Cho D, Yoon W-H, et al. Validation of progression-free survival rate at 6 months and objective response rate for estimating overall survival in immune checkpoint inhibitor trials: a systematic review and meta-analysis. *JAMA Netw Open*. 2020;3(9):e2011809. doi:10.1001/jamanetworkopen.2020.11809

eTable 1. Predictive Models Developed From Training Data Set to Estimate End Points

eTable 2. Summary of Included Trials

eTable 3. Summary of Immune Checkpoint Inhibitor Arms in Included Trials

eFigure 1. Six-Month Progression Free Survival Model–Predicted vs Observed 12-Month Overall Survival in Immune Checkpoint Inhibitor Arms in the Subgroups and Validation Trial Data Set

eFigure 2. Objective Response Rate Model–Predicted vs Observed 12-Month Overall Survival in Immune Checkpoint Inhibitor Arms in the Subgroups and Validation Trial Data Set

eFigure 3. Correlations Between Objective Response Rate and 12-Month Overall Survival in Immune Checkpoint Inhibitor Arms in the Subgroups and Entire Trial Data Set

eReferences

This supplementary material has been provided by the authors to give readers additional information about their work.

© 2020 Kok P-S et al. *JAMA Network Open.*

eTable 1. Predictive Models Developed From Training Data Set to Estimate End Points

**Model from 20 RCTs**	**Model equation**
PFS6 to predict OS12	OS12 = (1.06 x PFS6) + 0.16 + (0.04 × melanoma) - (0.03 × NSCLC) + (0 × other tumors)
ORR to predict OS12	OS12 = (0.15 x ORR) + 0.52 + (0 × melanoma) - (0.02 × NSCLC) - (0.01 × other tumors)
**Refitted model from 60 RCTs**	
ORR to predict OS12	OS12 = (1.012324 x ORR) + 0.3006825 + (0 x melanoma) + (0.0021079 x NSCLC) – (0.038521 x other tumors)

Abbreviation: PFS6, 6-month progression-free survival rate; OS12, 12-month overall survival rate; ORR, objective response rate; NSCLC, non-small cell lung cancer

© 2020 Kok P-S et al. *JAMA Network Open.*

eTable 2. Summary of Included Trials

Trial	Treatment comparisons	Phase	Tumour	Line	Experimental arm	Selected PD-L1 population	Sample size of ICI arm
**Training dataset**							
Checkmate 017[1]	ICI vs C	3	NSCLC	2	Nivolumab	No	135
Checkmate 025[2]	ICI vs C	3	Renal	2	Nivolumab	No	410
Checkmate 037[3, 4]	ICI vs C	3	Melanoma	2	Nivolumab	No	272
Checkmate 057[5]	ICI vs C	3	NSCLC	2	Nivolumab	No	292
Checkmate 141[6]	ICI vs C	3	Head and Neck SCC	2	Nivolumab	No	240
Checkmate 066[7]	ICI-C vs C	3	Melanoma	1	Nivolumab + Dacarbazine	No	210
Checkmate 026[8]	ICI vs C	3	NSCLC	1	Nivolumab	Yes	271
Attraction2[9]	ICI vs Placebo	3	Gastric/ Oesophageal	3	Nivolumab	No	330
CA184-024[10]	ICI-C vs C	3	Melanoma	1	Ipilimumab + Dacarbazine, followed by Dacarbazine, followed by Ipilimumab	No	250
CA 184-095[11]	ICI vs Placebo	3	Prostate	1	Ipilimumab	No	400
Keynote 010[12]	ICI vs C	2/3	NSCLC	2	Pembrolizumab	Yes	346
Keynote 010[12]	ICI vs C	2/3	NSCLC	2	Pembrolizumab	Yes	345
Keynote 024[13, 14]	ICI vs C	3	NSCLC	1	Pembrolizumab	No	154
Keynote 002[15, 16]	ICI vs C	2	Melanoma	3	Pembrolizumab	No	181
Keynote 002[15, 16]	ICI vs C	2	Melanoma	3	Pembrolizumab	No	180
Keynote 021[17, 18]	ICI-C vs C	2	NSCLC	1	Pembrolizumab + Carboplatin + Pemetrexed, followed by Pembrolizumab + Pemetrexed	No	60
OAK[19]	ICI vs C	3	NSCLC	2	Atezolizumab	No	425
Poplar[20]	ICI vs C	2	NSCLC	2	Atezolizumab	No	144
NCT00257205[21]	ICI vs C	3	Melanoma	1	Tremelimumab	No	328

© 2020 Kok P-S et al. *JAMA Network Open.*

DETERMINE[22]	ICI vs Placebo	2	Malignant Mesothelioma	2	Tremelimumab	No	382
CA 184-041[23]	ICI-C vs C	2	NSCLC	1	Carboplatin + Paclitaxel, followed by Ipilimumab + Carboplatin + Paclitaxel, followed by Ipilimumab	No	68
CA 184-041[23]	ICI-C vs C	2	NSCLC	1	Ipilimumab+ Carboplatin+ Paclitaxel, followed by Carboplatin + Paclitxel, followed by Ipilimumab	No	70
CA 184-041[16]	ICI-C vs C	2	SCLC	1	Carboplatin + Paclitaxel, followed by Ipilimumab + Carboplatin + Paclitaxel, followed by Ipilimumab	No	45
CA 184-041[24]	ICI-C vs C	2	SCLC	1	Ipilimumab+ Carboplatin+ Paclitaxel, followed by Carboplatin + Paclitxel, followed by Ipilimumab	No	45
CA 184-056[25]	ICI-C vs C	3	SCLC	1	Ipilimumab + Carboplatin/ Cisplatin and Etoposide, followed by Ipilimumab	No	566
**Validation dataset**							
IMPower 150[26]	ICI-C vs C	3	NSCLC	1	Atezolizumab + Bevacizumab + Paclitaxel + Carboplatin	No	356
Keynote 189[27]	ICI-C vs C	3	NSCLC	1	Pembrolizumab + Platinum + Pemetrexed	No	410
Keynote 407[28]	ICI-C vs C	3	NSCLC	1	Pembrolizumab+ Carboplatin + Paclitaxel/ nab-paclitaxel, followed by Pembrolizumab	No	278
Checkmate 227[29]	ICI-C vs C	3	NSCLC	1	Nivolumab + Chemotherapy	No	177
IMPassion 130[30]	ICI-C vs C	3	Triple negative breast cancer	1	Atezolizumab + nab-paclitaxel	Yes	451
IMPower 133[31]	ICI-C vs C	3	SCLC	1	Atezolizumab + Carboplatin + Etoposide	No	201
Keynote 048[32]	ICI-C vs C	3	Head and Neck SCC	1	Pembrolizumab + Carboplatin/ Cisplatin + 5FU, followed by Pembrolizumab	No	281
IMPower 131[33]	ICI-C vs C	3	NSCLC	1	Atezolizumab+ Carboplatin + nab-paclitaxel	No	343
IMPower 132[34]	ICI-C vs C	3	NSCLC	1	Atezolizumab + Cisplatin/ Carboplatin + Pemetrexed, followed by Atezolizumab + Pemetrexed	No	292
Kate2[35]	ICI-C vs C	2	Triple negative breast cancer	2+	Atezolizumab + TDM1	No	133
Keynote 062[36]	ICI-C vs C	3	Gastric/ Oesophageal	1	Pembrolizumab+ Chemotherapy (Cisplatin + 5FU/ Capecitabine)	Yes	257

© 2020 Kok P-S et al. *JAMA Network Open.*

IMpower 130[37]	ICI-C vs C	3	NSCLC	1	Atezolizumab + Carboplatin + nab-paclitaxel, followed by Atezolizumab	No	451
Javelin Renal 101[38]	ICI-TKI vs TKI	3	Renal	1	Avelumab + Axitinib	Yes	270
Keynote 426[21]	ICI-TKI vs TKI	3	Renal	1	Pembrolizumab +Axitinib	No	432
ImBlaze370[39]	ICI-TKI vs TKI	3	CRC	3	Atezolizumab + Cobimetinib	No	183
CCTG CO.26[39]	ICI vs BSC	2	CRC	3+	Durvalumab + Tremelimumab, followed by Durvalumab	No	119
Keynote 240[40]	ICI vs BSC	3	HCC	2	Pembrolizumab + BSC	No	278
Checkmate 227[41]	ICI vs C	3	NSCLC	1	Ipilimumab + Nivolumab	Yes	139
Keynote 048[32]	ICI vs C	3	Head and Neck SCC	1	Pembrolizumab	No	301
Javelin Lung 200[42]	ICI vs C	3	NSCLC	2	Avelumab	Yes	264
ARCTIC[43]	ICI vs C	3	NSCLC	3	Durvalumab	Yes	62
ARCTIC[43]	ICI vs C	3	NSCLC	3	Durvalumab + Tremelimumab, followed by Durvalumab	Yes	174
Checkmate 214[44]	ICI vs C	3	Renal	1	Ipilimumab + Nivolumab, followed by Nivolumab	No	425
IMMotion 151[45]	ICI vs C	3	Renal	1	Atezolizumab + Bevacizumab	Yes	178
Keynote 040[46]	ICI vs C	3	Head and Neck SCC	2	Pembrolizumab	Yes	247
Javelin Gastric 300[47]	ICI vs C	3	Gastric/ Oesophageal	3	Avelumab	No	185
IMvigor 211[48]	ICI vs C	3	Urothelial	2	Atezolizumab	Yes	116
IMMotion 150[49]	ICI vs C	2	Renal	1	Atezolizumab	Yes	103
IMMotion 150[49]	ICI vs C	2	Renal	1	Atezolizumab + Bevacizumab	Yes	101
Checkmate 331[50]	ICI vs C	3	SCLC	2	Nivolumab	No	284
Mystic[51]	ICI vs C	3	NSCLC	1	Durvalumab + Tremelimumab	Yes	163
Mystic[51]	ICI vs C	3	NSCLC	1	Durvalumab	Yes	163
Keynote 181[52, 53]	ICI vs C	3	Gastric/ Oesophageal	2	Pembrolizumab	Yes	107

© 2020 Kok P-S et al. *JAMA Network Open.*

Keynote 061[54]	ICI vs C	3	Gastric/ Oesophageal	2	Pembrolizumab	Yes	196
Keynote 042[55]	ICI vs C	3	NSCLC	1	Pembrolizumab	Yes	637
IMBlaze370[39]	ICI vs C	3	CRC	3	Atezolizumab	No	90
EAGLE[56]	ICI vs C	3	Head and Neck SCC	2+	Durvalumab	No	240
EAGLE[56]	ICI vs C	3	Head and Neck SCC	2+	Durvalumab + Tremelimumab	No	247
Keynote 062[36]	ICI vs C	3	Gastric/ Oesophageal	1	Pembrolizumab	Yes	256
Checkmate 078[57]	ICI vs C	3	NSCLC	2	Nivolumab	No	338
Keynote 045[58]	ICI vs C	3	Urothelial	2	Pembrolizumab	Yes	270
Checkmate 067[59]	ICI vs ICI	3	Melanoma	1	Ipilimumab + Nivolumab, followed by Nivolumab	No	314
Checkmate 067[59]	ICI vs ICI	3	Melanoma	1	Nivolumab	No	316
NCT01927419[60]	ICI vs ICI	2	Melanoma	1	Ipilimumab + Nivolumab, followed by Nivolumab	No	72
Keynote 006[61]	ICI vs ICI	3	Melanoma	1	Pembrolizumab	No	279
Keynote 006[61]	ICI vs ICI	3	Melanoma	1	Pembrolizumab	No	277
NCT01740297[62]	ICI vs T-Vec	2	Melanoma	1-2	Ipilimumab + Talimogene laherparepvec	No	98
LUNG-MAP[63]	ICI vs ICI	3	NSCLC	1-3	Ipilimumab + Nivolumab	No	125
IFCT-1501 MAPS2[64]	ICI vs ICI	2	Malignant Mesothelioma	2+	Nivolumab	No	63

Abbreviations: ICI, immune checkpoint inhibitor; C, Chemotherapy; ICI-C, combination of immune checkpoint inhibitor and chemotherapy; ICI-TKI, combination of immune checkpoint inhibitor and tyrosine kinase inhibitor; BSC, best supportive care; PD-L1, programmed death-ligand 1; NSCLC, non-small cell lung cancer; SCLC, small cell lung cancer; CRC, colorectal cancer; SCC, squamous cell carcinoma.

© 2020 Kok P-S et al. *JAMA Network Open.*

eTable 3. Summary of Immune Checkpoint Inhibitor Arms in Included Trials

**Summary of ICI arms**	**Training dataset (n=25)**	**Validation dataset (n=49)**	**Combined dataset (n=74)**
**Phase**			
3	14 (56)_	42 (86)	56 (76)
2 or 2/3	11 (44)	7 (14)	18 (24)
**ICI regimen**			
ICI combined with chemotherapy or TKI	8 (32)	15 (30)	23 (31)
ICI-only (singlet/ doublet)	17 (68)	34 (70)	51 (69)
**Line of treatment**			
First line	12 (48)	28 (57)	40 (54)
Second and subsequent line	13 (52)	21 (43)	34 (46)
**Tumour**			
Melanoma	6 (24)	6 (12)	12 (16)
NSCLC	11 (44)	16 (33)	27 (36)
Others	8 (32)	27 (55)	35 (47)
**PD-L1 selection**			
PD-L1 selected	3 (12)	19 (39)	22 (30)
PD-L1 unselected	22 (88)	30 (61)	52 (70)

Note. Values are No. (%) unless otherwise indicated.

Abbreviation: ICI, immune checkpoint inhibitor; PD-L1, programmed death-ligand 1; NSCLC, non-small cell lung cancer

© 2020 Kok P-S et al. *JAMA Network Open.*

eFigure 1. Six-Month Progression Free Survival Model–Predicted vs Observed 12-Month Overall Survival in Immune Checkpoint Inhibitor Arms in the Subgroups and Validation Trial Data Set

A. ICI-only

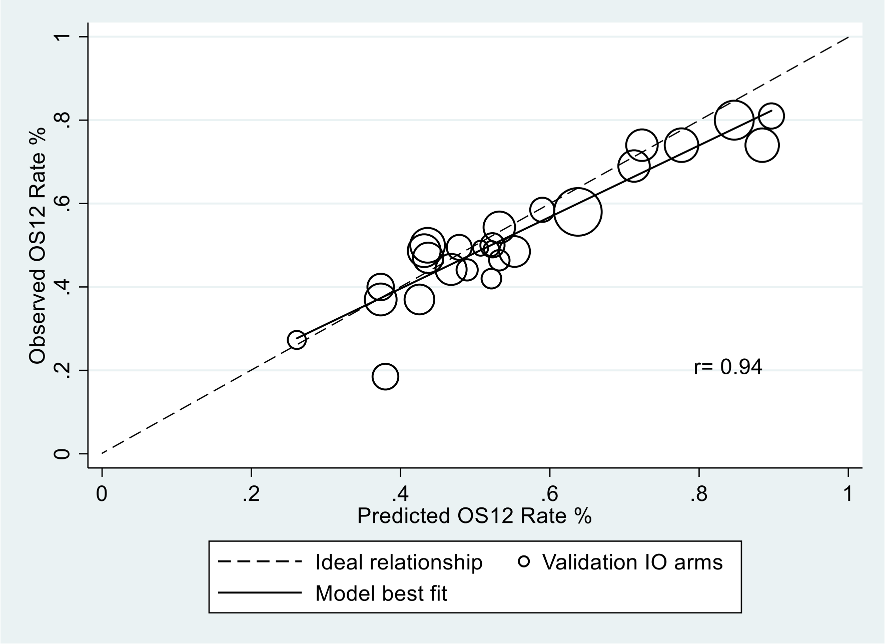

B. ICI-C

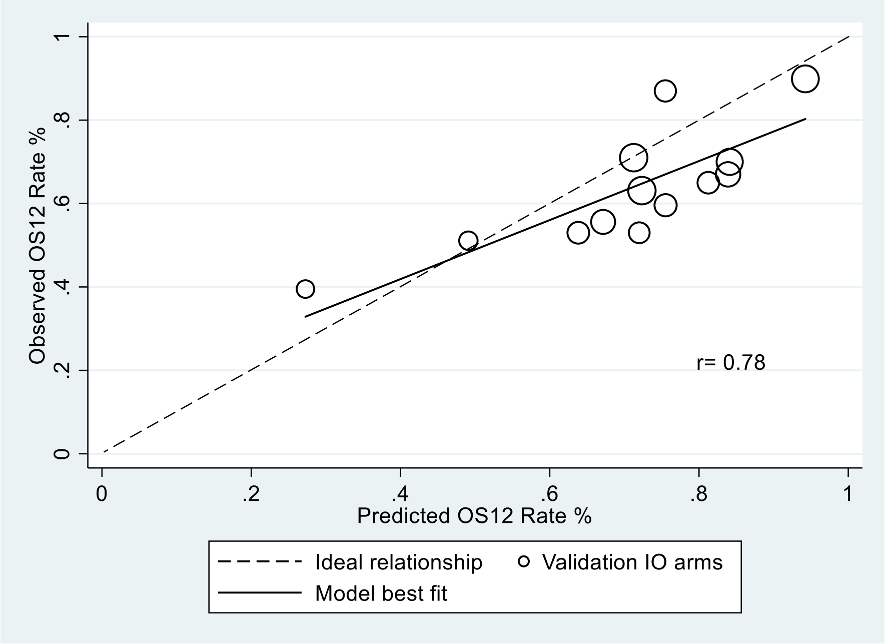

© 2020 Kok P-S et al. *JAMA Network Open.*

C. First line

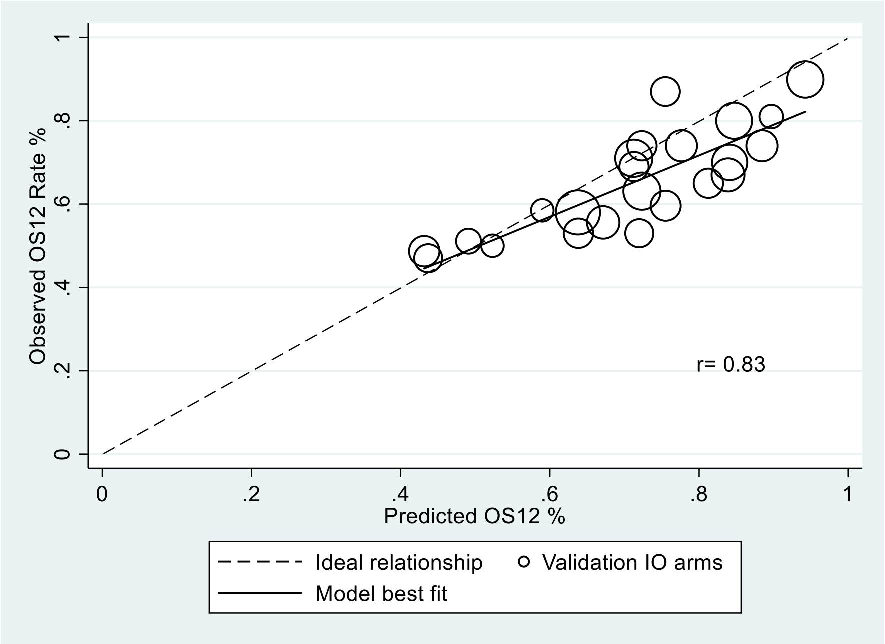

D. Second and subsequent line

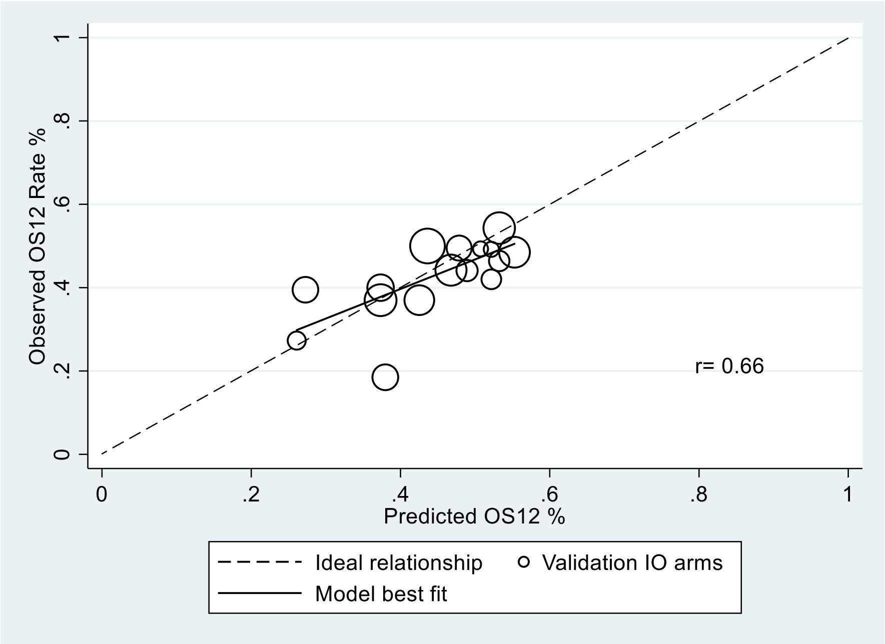

© 2020 Kok P-S et al. *JAMA Network Open.*

E. PD-L1 selected

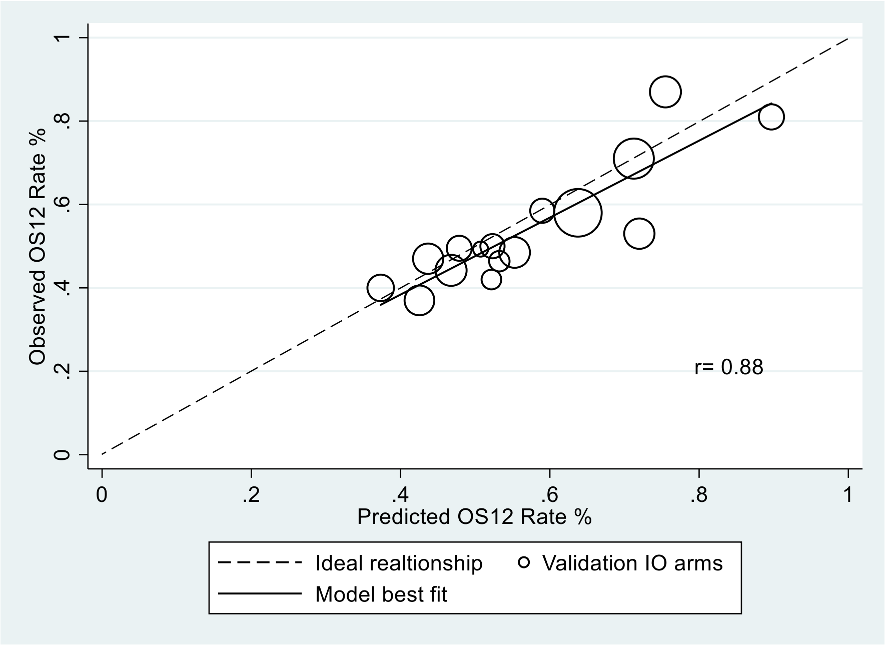

F. PD-L1 unselected

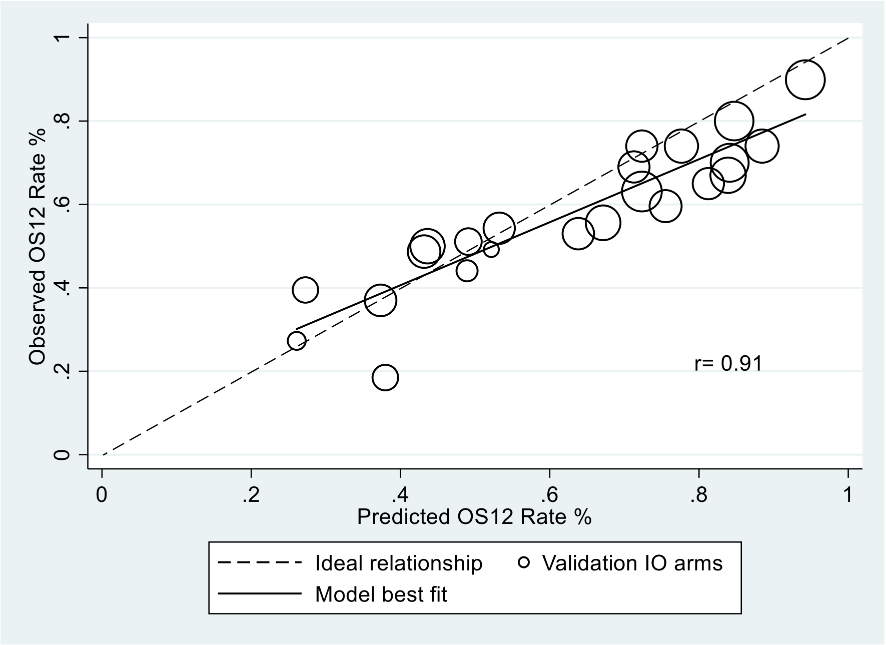

A: ICI-only B: ICI-C C: First line. D: Second and subsequent line. E. PD-L1 selected. F. PD-L1 unselected. Size of each circle represents size of immune checkpoint inhibitor arm.

Abbreviations: PFS6, 6-month progression-free survival; OS, overall survival; OS12, 12-month overall survival rate; ICI, immune checkpoint inhibitor; ICI-C, combination of immune checkpoint inhibitor and chemotherapy or tyrosine kinase inhibitor; PD-L1, programmed death-ligand 1

© 2020 Kok P-S et al. *JAMA Network Open.*

eFigure 2. Objective Response Rate Model–Predicted vs Observed 12-Month Overall Survival in Immune Checkpoint Inhibitor Arms in the Subgroups and Validation Trial Data Set

A. ICI-only

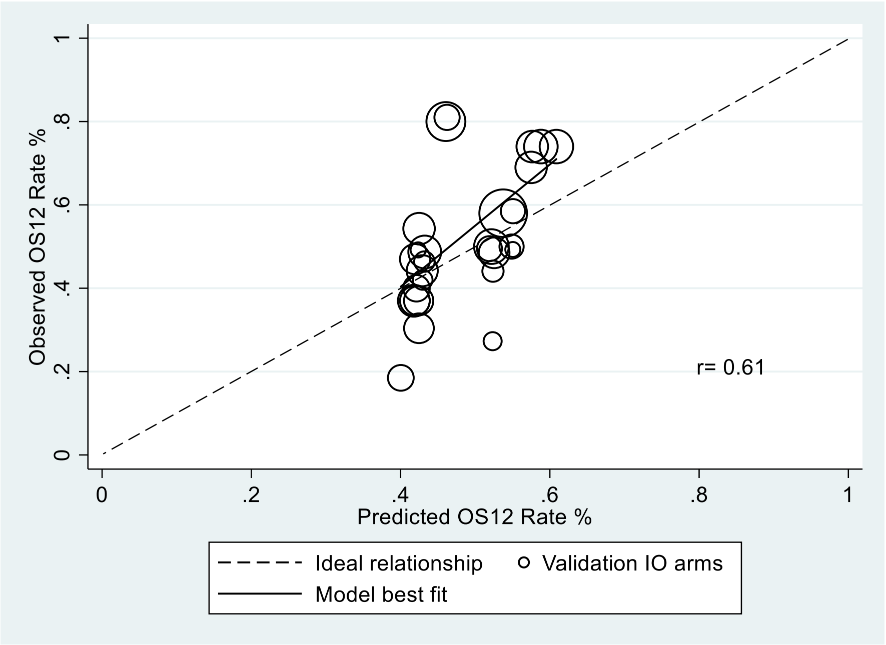

B. ICI-C

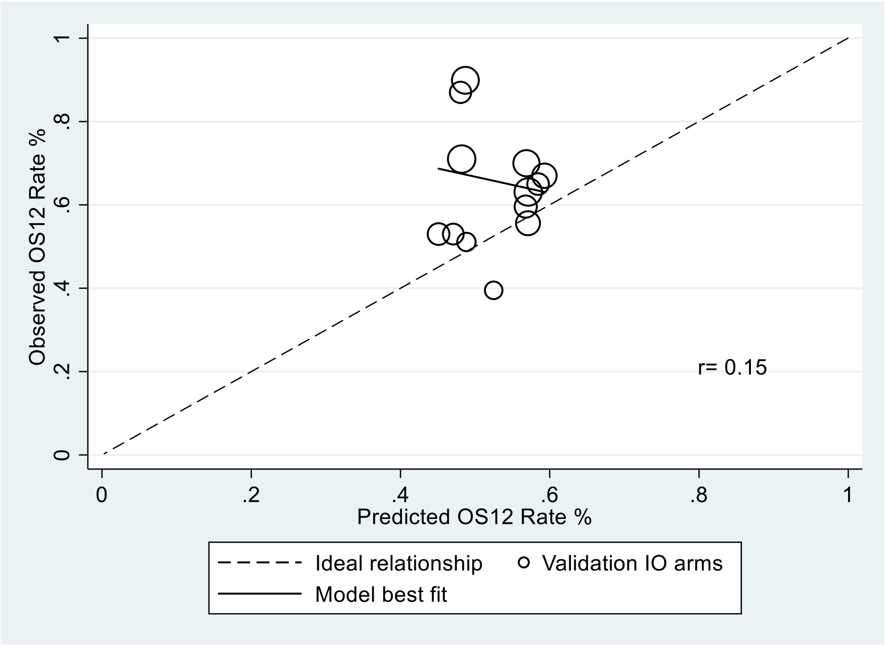

© 2020 Kok P-S et al. *JAMA Network Open.*

C. First line

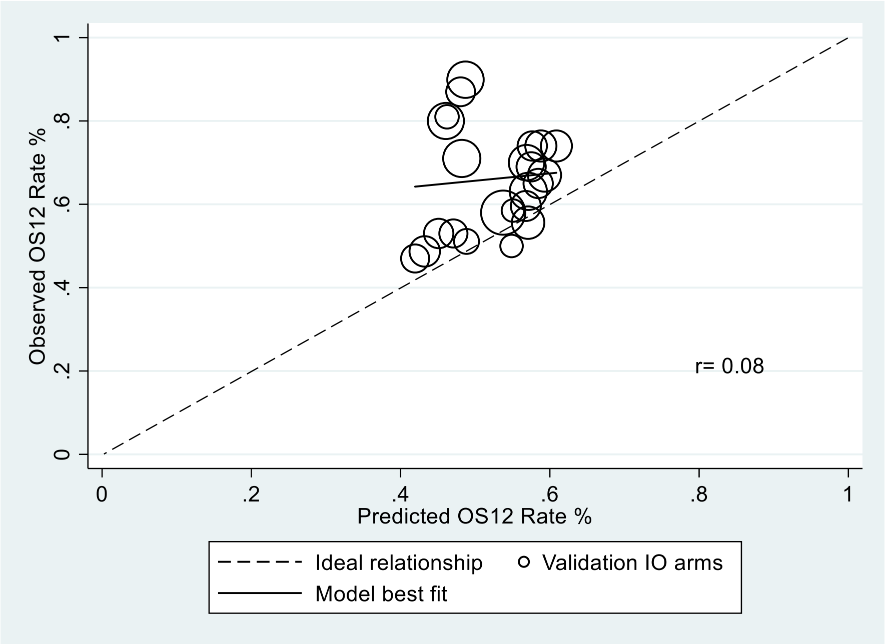

D. Second and subsequent line

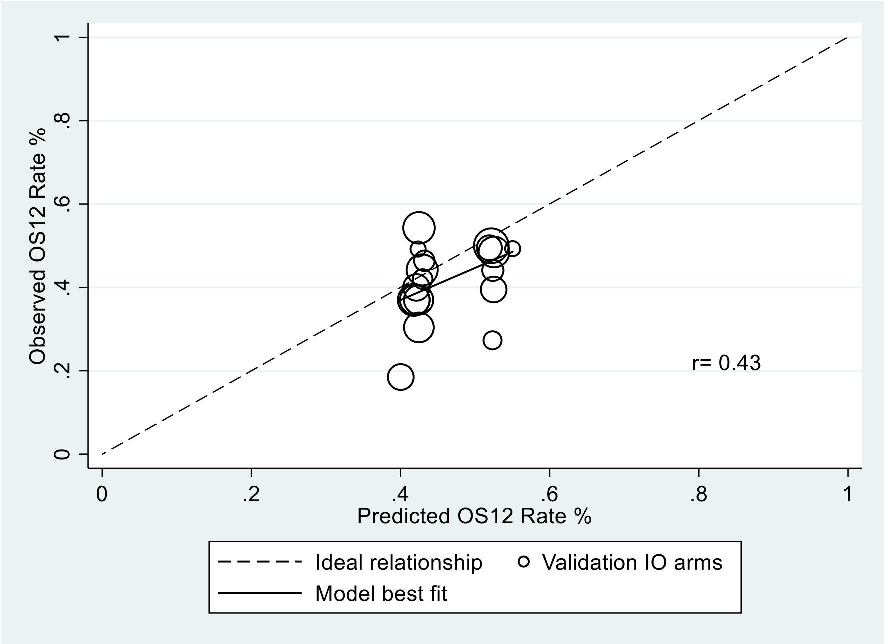

© 2020 Kok P-S et al. *JAMA Network Open.*

E. PD-L1 selected

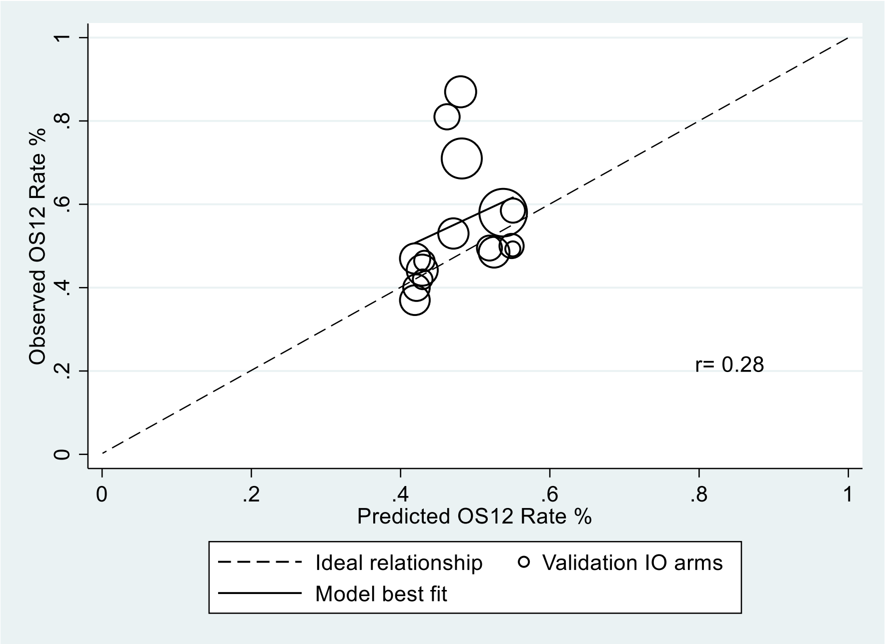

F. PD-L1 unselected

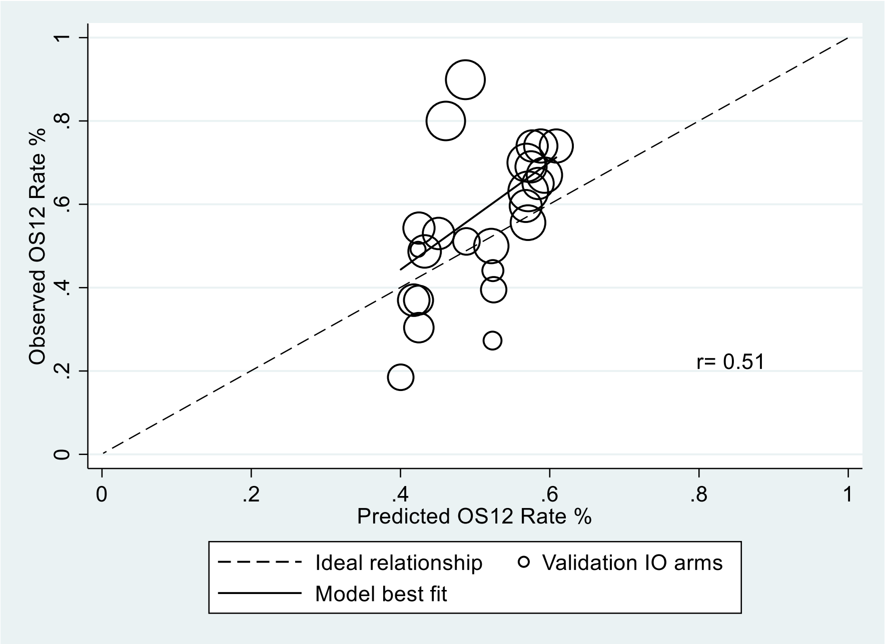

A: ICI-only B: ICI-C C: First line D: Second and subsequent line E. PD-L1 selected F. PD-L1 unselected. Size of each circle represents size of immune checkpoint inhibitor arm.

Abbreviations: ORR, objective response rate; OS, overall survival; OS12, 12-month overall survival rate; ICI, immune checkpoint inhibitor; ICI-C, combination of immune checkpoint inhibitor and chemotherapy or tyrosine kinase inhibitor; PD-L1, programmed death-ligand 1.

© 2020 Kok P-S et al. *JAMA Network Open.*

eFigure 3. Correlations Between Objective Response Rate and 12-Month Overall Survival in Immune Checkpoint Inhibitor Arms in the Subgroups and Entire Trial Data Set

A. ICI-only

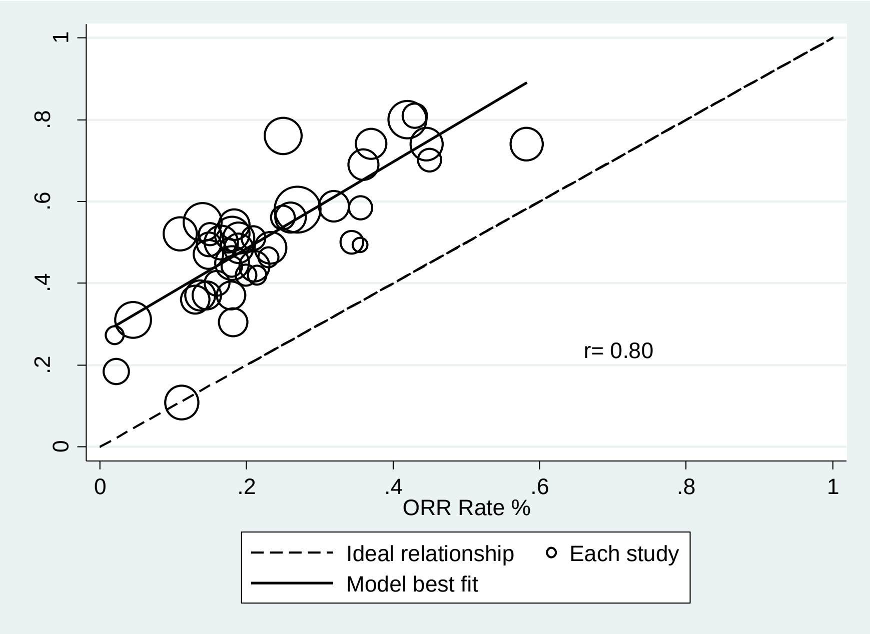

B. ICI-C

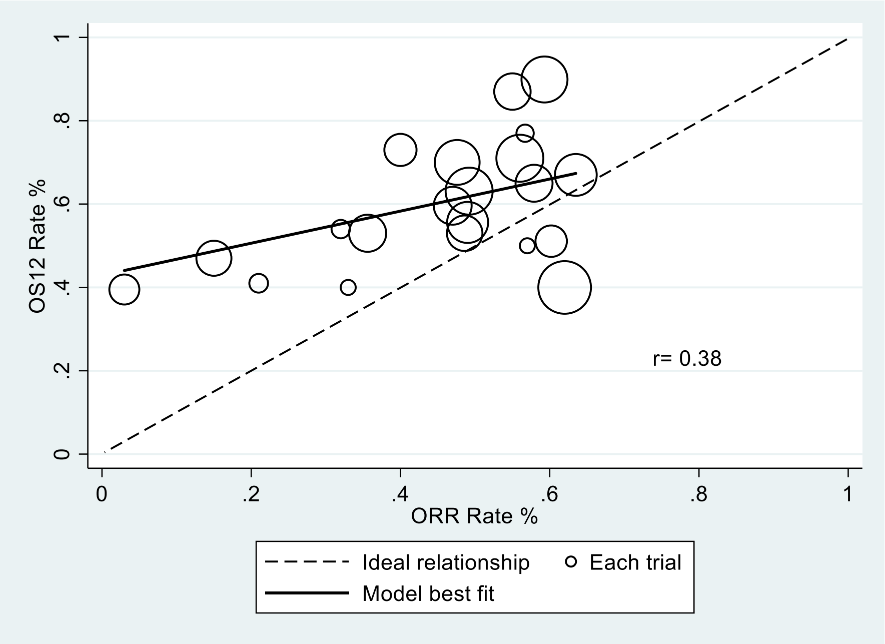

© 2020 Kok P-S et al. *JAMA Network Open.*

C. Second and subsequent line

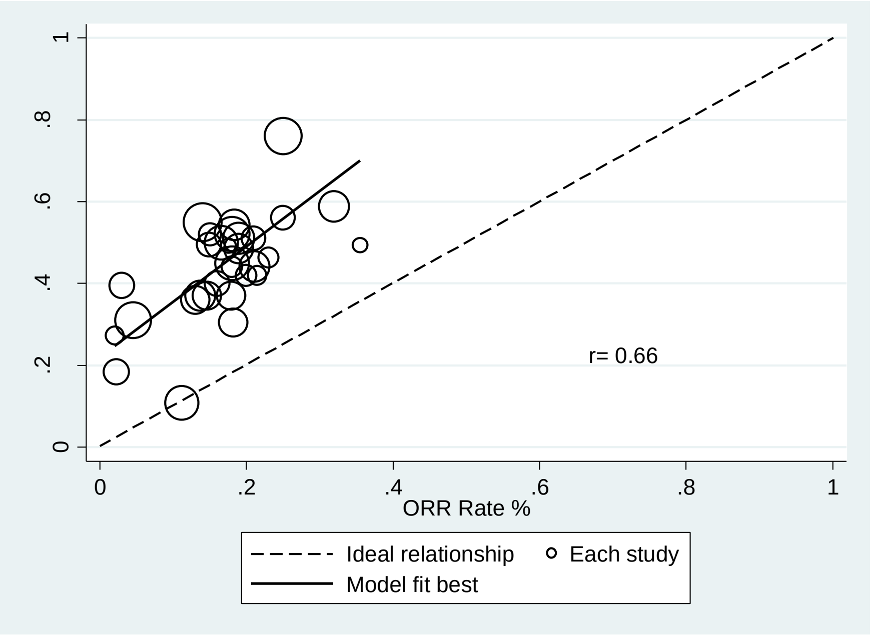

D. First line

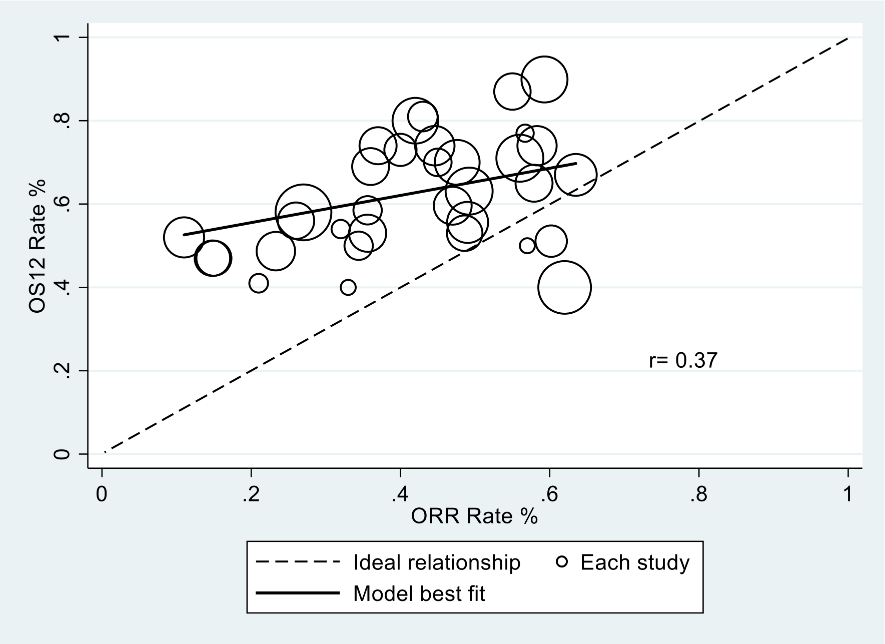

A: ICI-only B: ICI-C C: First line D: Second and subsequent line. Size of each circle represents size of immune checkpoint inhibitor arm.

Abbreviations: ORR, objective response rate; OS, overall survival; OS12, 12-month overall survival rate; ICI, immune checkpoint inhibitor; ICI-C, combination of immune checkpoint inhibitor and chemotherapy or tyrosine kinase inhibitor.

© 2020 Kok P-S et al. *JAMA Network Open.*

eReferences.

1. Brahmer J, et al. Nivolumab versus Docetaxel in Advanced Squamous-Cell Non–Small-Cell Lung Cancer. New England Journal of Medicine. 2015;373:123-35.
2. Motzer RJ, et al. Nivolumab versus Everolimus in Advanced Renal-Cell Carcinoma. New England Journal of Medicine. 2015;373:1803-13.
3. Weber JS, et al. Nivolumab versus chemotherapy in patients with advanced melanoma who progressed after anti-CTLA-4 treatment (CheckMate 037): a randomised, controlled, open-label, phase 3 trial. The Lancet Oncology. 2015;16:375-84.
4. Larkin J, et al. Overall Survival in Patients With Advanced Melanoma Who Received Nivolumab Versus Investigator's Choice Chemotherapy in CheckMate 037: A Randomized, Controlled, Open-Label Phase III Trial. J Clin Oncol. 2018;36:383-90.
5. Borghaei H, et al. Nivolumab versus Docetaxel in Advanced Nonsquamous Non–Small-Cell Lung Cancer. New England Journal of Medicine. 2015;373:1627-39.
6. Ferris RL, et al. Nivolumab for Recurrent Squamous-Cell Carcinoma of the Head and Neck. New England Journal of Medicine. 2016;375:1856-67.
7. Robert C, et al. Nivolumab in Previously Untreated Melanoma without BRAF Mutation. New England Journal of Medicine. 2014;372:320-30.
8. Carbone DP, et al. First-Line Nivolumab in Stage IV or Recurrent Non-Small-Cell Lung Cancer. N Engl J Med. 2017;376:2415-26.
9. Kang YK, et al. Nivolumab in patients with advanced gastric or gastro-oesophageal junction cancer refractory to, or intolerant of, at least two previous chemotherapy regimens (ONO-4538-12, ATTRACTION-2): a randomised, double-blind, placebo-controlled, phase 3 trial. Lancet. 2017;390:2461-71.
10. Robert C, et al. Ipilimumab plus Dacarbazine for Previously Untreated Metastatic Melanoma. New England Journal of Medicine. 2011;364:2517-26.
11. Beer TM, et al. Randomized, Double-Blind, Phase III Trial of Ipilimumab Versus Placebo in Asymptomatic or Minimally Symptomatic Patients With Metastatic Chemotherapy-Naive Castration-Resistant Prostate Cancer. J Clin Oncol. 2017;35:40-7.
12. Herbst RS, et al. Pembrolizumab versus docetaxel for previously treated, PD-L1-positive, advanced non-small-cell lung cancer (KEYNOTE-010): a randomised controlled trial. The Lancet. 2016;387:1540-50.
13. Reck M, et al. Pembrolizumab versus Chemotherapy for PD-L1-Positive Non-Small-Cell Lung Cancer. N Engl J Med. 2016;375:1823-33.
14. Reck M, et al. Updated Analysis of KEYNOTE-024: Pembrolizumab Versus Platinum-Based Chemotherapy for Advanced Non-Small-Cell Lung Cancer With PD-L1 Tumor Proportion Score of 50% or Greater. J Clin Oncol. 2019;37:537-46.
15. Ribas A, et al. Pembrolizumab versus investigator-choice chemotherapy for ipilimumab-refractory melanoma (KEYNOTE-002): a randomised, controlled, phase 2 trial. The Lancet Oncology. 2015;16:908-18.
16. Hamid O, et al. Final analysis of a randomised trial comparing pembrolizumab versus investigator-choice chemotherapy for ipilimumab-refractory advanced melanoma. Eur J Cancer. 2017;86:37-45.
17. Langer CJ, et al. Carboplatin and pemetrexed with or without pembrolizumab for advanced, non-squamous non-small-cell lung cancer: a randomised, phase 2 cohort of the open-label KEYNOTE-021 study. The Lancet Oncology. 2016;17:1497-508.
18. Borghaei H, et al. 24-Month Overall Survival from KEYNOTE-021 Cohort G: Pemetrexed and Carboplatin with or without Pembrolizumab as First-Line Therapy for Advanced Nonsquamous Non–Small Cell Lung Cancer. Journal of Thoracic Oncology. 2019;14:124-9.

© 2020 Kok P-S et al. *JAMA Network Open.*

19. Rittmeyer A, et al. Atezolizumab versus docetaxel in patients with previously treated non-small-cell lung cancer (OAK): a phase 3, open-label, multicentre randomised controlled trial. The Lancet. 2017;389:255-65.
20. Fehrenbacher L, et al. Atezolizumab versus docetaxel for patients with previously treated non-small-cell lung cancer (POPLAR): a multicentre, open-label, phase 2 randomised controlled trial. The Lancet. 2016;387:1837-46.
21. Ribas A, et al. Phase III randomized clinical trial comparing tremelimumab with standard-of-care chemotherapy in patients with advanced melanoma. J Clin Oncol. 2013;31:616-22.
22. Maio M, et al. Tremelimumab as second-line or third-line treatment in relapsed malignant mesothelioma (DETERMINE): a multicentre, international, randomised, double-blind, placebo-controlled phase 2b trial. The Lancet Oncology. 2017;18:1261-73.
23. Lynch TJ, et al. Ipilimumab in combination with paclitaxel and carboplatin as first-line treatment in stage IIIB/IV non-small-cell lung cancer: results from a randomized, double-blind, multicenter phase II study. J Clin Oncol. 2012;30:2046-54.
24. Reck M, et al. Ipilimumab in combination with paclitaxel and carboplatin as first-line therapy in extensive-disease-small-cell lung cancer: results from a randomized, double-blind, multicenter phase 2 trial. Ann Oncol. 2013;24:75-83.
25. Reck M, et al. Pembrolizumab versus Chemotherapy for PD-L1–Positive Non–Small-Cell Lung Cancer. New England Journal of Medicine. 2016;375:1823-33.
26. Socinski MA, et al. Atezolizumab for First-Line Treatment of Metastatic Nonsquamous NSCLC. New England Journal of Medicine. 2018;378:2288-301.
27. Gandhi L, et al. Pembrolizumab plus Chemotherapy in Metastatic Non–Small-Cell Lung Cancer. New England Journal of Medicine. 2018;378:2078-92.
28. Paz-Ares L, et al. Pembrolizumab plus Chemotherapy for Squamous Non–Small-Cell Lung Cancer. New England Journal of Medicine. 2018;379:2040-51.
29. Borghaei H, et al. Nivolumab (Nivo) + platinum-doublet chemotherapy (Chemo) vs chemo as first-line (1L) treatment (Tx) for advanced non-small cell lung cancer (NSCLC) with <1% tumor PD-L1 expression: Results from CheckMate 227. Journal of Clinical Oncology. 2018;36:9001-.
30. Schmid P, et al. Atezolizumab and Nab-Paclitaxel in Advanced Triple-Negative Breast Cancer. New England Journal of Medicine. 2018;379:2108-21.
31. Horn L, et al. First-Line Atezolizumab plus Chemotherapy in Extensive-Stage Small-Cell Lung Cancer. New England Journal of Medicine. 2018;0:null.
32. Rischin D, et al. Protocol-specified final analysis of the phase 3 KEYNOTE-048 trial of pembrolizumab (pembro) as first-line therapy for recurrent/metastatic head and neck squamous cell carcinoma (R/M HNSCC). Journal of Clinical Oncology. 2019;37:6000-.
33. Jotte RM, et al. IMpower131: Primary PFS and safety analysis of a randomized phase III study of atezolizumab + carboplatin + paclitaxel or nab-paclitaxel vs carboplatin + nab-paclitaxel as 1L therapy in advanced squamous NSCLC. Journal of Clinical Oncology. 2018;36:LBA9000-LBA.
34. Papadimitrakopoulou V, et al. OA05.07 IMpower132: PFS and Safety Results with 1L Atezolizumab + Carboplatin/Cisplatin + Pemetrexed in Stage IV Non-Squamous NSCLC. Journal of Thoracic Oncology. 2018;13:S332-S3.
35. Emens L, et al. Abstract PD3-01: Results from KATE2, a randomized phase 2 study of atezolizumab (atezo)+trastuzumab emtansine (T-DM1) vs placebo (pbo)+T-DM1 in previously treated HER2+ advanced breast cancer (BC). Cancer Research. 2019;79:PD3-01-PD3-.

© 2020 Kok P-S et al. *JAMA Network Open.*

36. Tabernero J, et al. Pembrolizumab with or without chemotherapy versus chemotherapy for advanced gastric or gastroesophageal junction (G/GEJ) adenocarcinoma: The phase III KEYNOTE-062 study. Journal of Clinical Oncology. 2019;37:LBA4007-LBA.
37. West H, et al. Atezolizumab in combination with carboplatin plus nab-paclitaxel chemotherapy compared with chemotherapy alone as first-line treatment for metastatic non-squamous non-small-cell lung cancer (IMpower130): a multicentre, randomised, open-label, phase 3 trial. Lancet Oncol. 2019;20:924-37.
38. Motzer RJ, et al. Avelumab plus Axitinib versus Sunitinib for Advanced Renal-Cell Carcinoma. New England Journal of Medicine. 2019;380:1103-15.
39. Eng C, et al. Atezolizumab with or without cobimetinib versus regorafenib in previously treated metastatic colorectal cancer (IMblaze370): a multicentre, open-label, phase 3, randomised, controlled trial. The Lancet Oncology. 2019;20:849-61.
40. Finn RS, et al. Results of KEYNOTE-240: phase 3 study of pembrolizumab (Pembro) vs best supportive care (BSC) for second line therapy in advanced hepatocellular carcinoma (HCC). Journal of Clinical Oncology. 2019;37:4004-.
41. Hellmann MD, et al. Nivolumab plus Ipilimumab in Lung Cancer with a High Tumor Mutational Burden. New England Journal of Medicine. 2018;378:2093-104.
42. Barlesi F, et al. Avelumab versus docetaxel in patients with platinum-treated advanced non-small-cell lung cancer (JAVELIN Lung 200): an open-label, randomised, phase 3 study. The Lancet Oncology. 2018;19:1468-79.
43. Kowalski DM, et al. 1378OARCTIC: Durvalumab + tremelimumab and durvalumab monotherapy vs SoC in ⩾ 3L advanced NSCLC treatment. Annals of Oncology. 2018;29.
44. Motzer RJ, et al. Nivolumab plus Ipilimumab versus Sunitinib in Advanced Renal-Cell Carcinoma. New England Journal of Medicine. 2018;378:1277-90.
45. Motzer RJ, et al. IMmotion151: A Randomized Phase III Study of Atezolizumab Plus Bevacizumab vs Sunitinib in Untreated Metastatic Renal Cell Carcinoma (mRCC). Journal of Clinical Oncology. 2018;36:578-.
46. Cohen EEW, et al. Pembrolizumab versus methotrexate, docetaxel, or cetuximab for recurrent or metastatic head-and-neck squamous cell carcinoma (KEYNOTE-040): a randomised, open-label, phase 3 study. The Lancet. 2019;393:156-67.
47. Bang YJ, et al. Phase III, randomised trial of avelumab versus physician's choice of chemotherapy as third-line treatment of patients with advanced gastric or gastro-oesophageal junction cancer: primary analysis of JAVELIN Gastric 300. Ann Oncol. 2018;29:2052-60.
48. Powles T, et al. Atezolizumab versus chemotherapy in patients with platinum-treated locally advanced or metastatic urothelial carcinoma (IMvigor211): a multicentre, open-label, phase 3 randomised controlled trial. The Lancet. 2018;391:748-57.
49. McDermott DF, et al. Clinical activity and molecular correlates of response to atezolizumab alone or in combination with bevacizumab versus sunitinib in renal cell carcinoma. Nat Med. 2018;24:749-57.
50. Reck M, et al. LBA5Efficacy and safety of nivolumab (nivo) monotherapy versus chemotherapy (chemo) in recurrent small cell lung cancer (SCLC): Results from CheckMate 331. Annals of Oncology. 2018;29.
51. Rizvi NA, et al. LBA6Durvalumab with or without tremelimumab vs platinum-based chemotherapy as first-line treatment for metastatic non-small cell lung cancer: MYSTIC. Annals of Oncology. 2018;29.
52. Kojima T, et al. Pembrolizumab versus chemotherapy as second-line therapy for advanced esophageal cancer: Phase III KEYNOTE-181 study. Journal of Clinical Oncology. 2019;37:2-.

© 2020 Kok P-S et al. *JAMA Network Open.*

53. Shah MA, et al. Pembrolizumab versus chemotherapy as second-line therapy for advanced esophageal cancer: Phase 3 KEYNOTE-181 study. Journal of Clinical Oncology. 2019;37:4010-.
54. Shitara K, et al. Pembrolizumab versus paclitaxel for previously treated, advanced gastric or gastro-oesophageal junction cancer (KEYNOTE-061): a randomised, open-label, controlled, phase 3 trial. Lancet. 2018;392:123-33.
55. Mok TSK, et al. Pembrolizumab versus chemotherapy for previously untreated, PD-L1-expressing, locally advanced or metastatic non-small-cell lung cancer (KEYNOTE-042): a randomised, open-label, controlled, phase 3 trial. Lancet. 2019;393:1819-30.
56. Licitra LF, et al. EAGLE: A phase 3, randomized, open-label study of durvalumab (D) with or without tremelimumab (T) in patients (pts) with recurrent or metastatic head and neck squamous cell carcinoma (R/M HNSCC). Journal of Clinical Oncology. 2019;37:6012-.
57. Wu YL, et al. Nivolumab Versus Docetaxel in a Predominantly Chinese Patient Population With Previously Treated Advanced NSCLC: CheckMate 078 Randomized Phase III Clinical Trial. J Thorac Oncol. 2019;14:867-75.
58. Bellmunt J, et al. Pembrolizumab as Second-Line Therapy for Advanced Urothelial Carcinoma. New England Journal of Medicine. 2017;376:1015-26.
59. Hodi FS, et al. Nivolumab plus ipilimumab or nivolumab alone versus ipilimumab alone in advanced melanoma (CheckMate 067): 4-year outcomes of a multicentre, randomised, phase 3 trial. Lancet Oncol. 2018;19:1480-92.
60. Postow MA, et al. Nivolumab and ipilimumab versus ipilimumab in untreated melanoma. N Engl J Med. 2015;372:2006-17.
61. Schachter J, et al. Pembrolizumab versus ipilimumab for advanced melanoma: final overall survival results of a multicentre, randomised, open-label phase 3 study (KEYNOTE-006). The Lancet. 2017;390:1853-62.
62. Chesney J, et al. Randomized, Open-Label Phase II Study Evaluating the Efficacy and Safety of Talimogene Laherparepvec in Combination With Ipilimumab Versus Ipilimumab Alone in Patients With Advanced, Unresectable Melanoma. J Clin Oncol. 2018;36:1658-67.
63. Bazhenova L, et al. A phase III randomized study of nivolumab plus ipilimumab versus nivolumab for previously treated patients with stage IV squamous cell lung cancer and no matching biomarker (Lung-MAP Sub-Study S1400I, NCT02785952). Journal of Clinical Oncology. 2019;37:9014-.
64. Scherpereel A, et al. Nivolumab or nivolumab plus ipilimumab in patients with relapsed malignant pleural mesothelioma (IFCT-1501 MAPS2): a multicentre, open-label, randomised, non-comparative, phase 2 trial. Lancet Oncol. 2019;20:239-53.

© 2020 Kok P-S et al. *JAMA Network Open.*
